# The Regenerative Potential of Facial Nerve Motoneurons following Chronic Axotomy in Rats

**DOI:** 10.1155/2020/8884511

**Published:** 2020-08-01

**Authors:** Yusu Ni, Diyan Chen, Yi Jiang, Danhong Qiu, Wen Li, Huawei Li

**Affiliations:** ^1^Otology and Skull Base Surgery Department, Eye and ENT Hospital of Shanghai Medical School, Fudan University, China; ^2^Department of Ophthalmology, Shanghai Xin Shi Jie Eye Hospital, Shanghai, China; ^3^Otolaryngology Department, Pudong Hospital, Shanghai, China; ^4^Central Laboratory, Eye and ENT Hospital of Shanghai Medical School, Fudan University, China; ^5^ENT Institute and Otorhinolaryngology Department of Eye & ENT Hospital, State Key Laboratory of Medical Neurobiology and MOE Frontiers Center for Brain Science, Fudan University, Shanghai 200031, China; ^6^Institutes of Biomedical Sciences, Fudan University, Shanghai 200032, China; ^7^NHC Key Laboratory of Hearing Medicine (Fudan University), Shanghai 200031, China; ^8^The Institutes of Brain Science and the Collaborative Innovation Center for Brain Science, Fudan University, Shanghai 200032, China

## Abstract

**Background:**

The precise mechanisms of nerve regeneration remain unclear. The potential of facial nerve regeneration and probable mechanisms involved following chronic facial nerve injury should be further studied.

**Methods:**

Adult male Wistar rats were used to model either (i) facial nerve injury (axotomy) or (ii) reinjury (chronic axotomy followed by a second axotomy within 5 months). The rats were housed in the animal facility of the Eye and ENT Hospital of Shanghai Medical School, Fudan University (Shanghai, China). Expression of Shh (sonic hedgehog) and growth-associated protein 43 (GAP43, a neuronal marker) was detected in bilateral facial nuclei using reverse transcriptase PCR, western blotting analysis, and immunohistochemistry. The number of surviving motoneurons was quantified, and facial nerve regeneration was examined using transmission electron microscopy.

**Results:**

Reinjury of the facial nerve 12 weeks after the first axotomy resulted in upregulation of GAP43 mRNA and protein expression in neurons ipsilateral to the axotomy; immunohistochemistry revealed that Shh expression was higher compared with control side facial nuclei at the same time point. GAP43 expression subsequently decreased.

**Conclusion:**

The greatest regeneration potential of the facial nerve occurred within 5 months following chronic axotomy in rats, and regeneration may involve the Shh signaling pathway.

## 1. Introduction

Peripheral facial paralysis was characterized by paralysis of all facial expression muscles in the affected side, and facial muscle movement disorder was the main characteristic, which caused great psychological stress, mental trauma to the patients. No matter what cause of peripheral facial paralysis, if the drug treatment was ineffective, they should consider early surgical treatment [1–4].

Although, some scholars believed the facial nerve had a greater capacity for regeneration than any other neuron in the central nervous system; in this regard, the facial nerve was very similar to peripheral motor nerves [5–8]. For those patients with facial paralysis for a long time, the curative effects after operation were often not ideal [1–4]; the most important reason was the loss of the most facial nerve motor neurons, which led to the ability decline of facial nerve regeneration [9].

For years, researchers have never stopped looking for effective treatments to promote facial nerve regeneration [10–13].

Previous studies have shown that nerve injuries induce a variety of molecular responses that may be involved in the regeneration of injured neurons [11, 13–16]. In the neurons, the efficacy and the specificity of neurotrophic factors to support regeneration depend on the presence of their respective receptors and their number. The receptors for NGF, FGF-2, BDNF, GDNF, and IGF-I are synthesized by neurons and are upregulated following axotomy. Seitz et al. research and analysis showed that recovery of motor function after peripheral nerve injury is related with a complex regulation of lesion-associated neurotrophic factors and cytokines, which include BDNF, FGF2, IGF2, IGF1, and NGF protein [17].

Some scholars have also made some progress in promoting the recovery of injured facial nerve function by using degradable neural catheters and dedifferentiated fat cells [18], either by local administration of nerve catheters (e.g., neurotrophic factors) [19] or by injecting stem cells into nerve ducts [20–23].

No matter which way to promote the regeneration of facial nerve after injury, how to protect or reduce the nonapoptosis of motor neurons of facial nerve after injury is indeed the most critical step to improve the repair of facial nerve regeneration [9]. Although many molecules involved in facial nerve repair have been characterized, the precise mechanisms of nerve regeneration remain unclear. Interestingly, some studies have demonstrated that electrical stimulation could promote peripheral nerve regeneration or the functional recovery of paralyzed facial nerves and nerve reinnervation of paralyzed muscles [24–26]. However, the mechanism by which electrical stimulation promotes nerve regeneration is unclear, and we speculate that it may be related to the electrical stimulation of the peripheral nerve, which activated the regenerative or functionally protective neural signaling pathway.

Mammals have three genes with homology to the Hh gene (sonic hedgehog (Shh), Indian hedgehog (Ihh), and desert hedgehog (Dhh)). Shh signaling played important roles for patterning and cell fate specification in the central nervous system, and Shh shows low expression in the neural stem/progenitor cells in the dorsal telencephalon. Shh signaling in neocortex development has been shown to regulate intermediate progenitor cells, thereby maintaining the proliferation, survival, and differentiation of neurons in the neocortex [27–30].

In adult rats, sonic hedgehog (Shh) expression is upregulated 24 hours after facial nerve axotomy and then starts to decline 4 weeks later [31]. Although the precise molecular circuitry of regeneration is unclear, this expression pattern implies a function for Shh in mature motoneurons [32].

In this study, we investigated the potential of facial nerve regeneration and whether it was affected by activation of the Shh signaling pathways.

## 2. Methods and Materials

### 2.1. Animals

Adult male Wistar rats (weighing 200–250 g) were housed in the animal facility of the Eye and ENT Hospital of Shanghai Medical School, Fudan University (Shanghai, China). All animal experiments and care protocols were performed under the approval of the institution's ethical committee for care and use of laboratory animals.

### 2.2. Axotomy Models

Animal experiments were performed under general anesthesia using an intraperitoneal injection of a mixture of ketamine hydrochloride (135 mg/kg) and xylazine hydrochloride (6.5 mg/kg). Animals were divided into two experimental groups. In Group I (axotomy), the right facial nerve stem (including the posterior auricular branch) was transected approximately 3 mm distal from the stylomastoid foramen, a 2 mm segment of the distal portion of the nerve was removed, the distal stump was ligated with 3-0 silk thread to prevent the regeneration of axons from their targets, and 5-0 silk thread was used to label the proximal nerve stump. In Group II (reinjury involving chronic axotomy followed by second axotomy), at 12, 20, 28, and 36 weeks (w) after the initial facial nerve axotomy, the proximal 1 mm nerve stump (including the posterior auricular branch) was reaxotomized and the distal stump was ligated with 3-0 silk thread. Intact contralateral sides served as controls. There were 10 rats in each experimental group at each observation time.

### 2.3. Tissue Collection

At 13, 21, 29, and 37 w after the initial facial nerve axotomy in Group I, and 1 w after the facial nerve was reaxotomized in Group II, ten rats were randomly selected. Five were transcardially perfused with normal saline (0.9% NaCl) followed by 4% paraformaldehyde (PFA). The brainstems of these rats were removed, postfixed in 4% PFA for 24 h, and dehydrated in phosphate-buffered saline (PBS) containing 15% sucrose followed by 30% sucrose/PBS solution. Tissues were then snap frozen and stored at -80°C. Coronal brainstem sections were cut at a thickness of 20 *μ*m with a cryostat and used for immunohistochemistry. Each treatment group was randomly examined to eliminate any systematic handling biases. The remaining five rats were rapidly decapitated under general anesthesia. The brains were removed quickly and stored in liquid nitrogen until required for reverse transcriptase PCR (RT-PCR) and western blotting analysis.

### 2.4. Immunohistochemistry

Cellular morphology was examined by staining slides with 1% toluidine blue (Sigma, St. Louis, MO). Briefly, slides were placed in distilled water for 2 min followed by 1% toluidine blue for 20 min at 40°C. Slides were then rinsed in water followed by 95% ethanol, and covered with a coverslip.

Immunohistochemistry was performed to identify cellular expression of growth-associated protein-43 (GAP43), Shh, and glial fibrillary acidic protein (GFAP). Double fluorescence labeling was performed to identify cellular expression of Shh in GAP43- or GFAP-positive cells using a mouse monoclonal anti-Shh antibody (Sigma, 1 : 1000 dilution) and polyclonal anti-GAP43 (rabbit anti-rat GAP43, 1 : 500 dilution; Abcam, Cambridge, UK) and anti-GFAP antibodies (rabbit anti-rat GFAP, 1 :  100 dilution; Sigma). Cryosections were fixed in 4% PFA containing 0.5% Triton X-100 before incubation with primary antibodies overnight at 4°C. Sections were then washed and incubated with a secondary fluorescein isothiocyanate- (FITC-) labeled antibody (goat anti-rabbit, 1 : 200 dilution; Jackson ImmunoResearch, West Grove, PA) or tetramethylrhodamine isothiocyanate- (TRITC-) labeled antibody (goat anti-mouse, 1 : 200 dilution; Jackson ImmunoResearch). Fluorescence images were captured using a confocal microscope (Leica, Wetzlar, Germany) and analyzed with Image Pro Plus software version 6.0 (Media Cybernetics, Rockville, MD).

### 2.5. Reverse Transcription Polymerase Chain Reaction (RT-PCR)

Frozen brain stems were quickly sectioned in a coronal orientation at a thickness of 100 *μ*m using a cryostat. Two sections of the experimental and control side facial nuclei at the same location (based on location within the brain stem) of each rat were homogenized in TRIzol reagent (Invitrogen, Carlsbad, CA). Total RNA was extracted and reverse transcribed using a SuperScript™ III First-Strand Synthesis System RT-PCR kit (Invitrogen). The PCR reaction contained 3 *μ*L of cDNA, 5 *μ*L of 10× PCR buffer, 1.5 *μ*L of 50 mM MgCl_2_, 1 *μ*L of 10 mM dNTP mixture (0.2 *μ*M each), 1 *μ*L of sense primer (0.2 *μ*M/L), 1 *μ*L of antisense primer (0.2 *μ*M/L), 0.2 *μ*L (1 unit) of Platinum Taq DNA Polymerase (Invitrogen), and H_2_O to generate a total volume of 50 *μ*L. GAP43 PCR reactions were performed using 5′-ATGCTGTGCTGTATGAGAAGAACC-3′ (sense) and 5′-GGCAACGTGGAAAGCCGTTTCTTAAAGT-3′ (antisense) primers [32] under the following conditions: 94°C for 2 min; 30 cycles of 94°C for 30 sec, 57°C for 30 sec, and 72°C for 45 sec; and final extension at 72°C for 10 min. GAPDH-specific primers [31] were 5′-TCGTGGAGTCTACTGGCGTCTT-3′ (sense) and 5′-CCTCTCTCTTGCTCTCAGTATC-3′ (antisense). GAPDH was used as an internal control. All primers were synthesized by Sangon Bio-Engineering Co. Ltd. (Shanghai, China). Amplification products and a 100 bp DNA ladder (Takara Bio, Kusatsu, Japan) were separated by 3% agarose gel electrophoresis and then visualized using ethidium bromide staining and ultraviolet light.

### 2.6. Western Blotting Analysis

Proteins were extracted using TRIzol reagent according to the manufacturer's instructions. Protein concentrations were measured using BCA Protein Assay Kit (Bipec Biopharma Corporation, USA) with bovine serum albumin standards and then equalized. Samples were denatured at 100°C for 5 min, separated by 12% sodium dodecyl sulfate polyacrylamide gel electrophoresis, and transferred to a 0.45 *μ*m polyvinylidene difluoride membrane (Immobilon-P; EMD Millipore, Burlington, MA). The membrane was blocked in a solution of 50 mM Tris HCl, 100 mM NaCl, and 0.1% Tween-20, pH 7.4 (TBST) containing 5% nonfat dry milk, followed by incubation with a 1 : 500 dilution of polyclonal rabbit anti-rat GAP43 antibody (Abcam) in 5% nonfat dry milk (in TBST) at 4°C overnight. Membranes were washed three times with TBST buffer for 5 min each and further incubated with a 1 : 2000 dilution of horseradish peroxidase- (HRP-) conjugated goat anti-rabbit IgG at room temperature for 2 h. After washing the membrane, HRP activity was detected using an enhanced chemiluminescence kit (Roche Diagnostics, Mannheim, Germany). GAPDH (1 : 5000, mouse anti-GAPDH; Kangcheng, Shanghai, China) was used as an internal control. X-ray autoradiography was performed using Kodak X-Omat BT film (Rochester, NY).

### 2.7. Facial Nerve Stem Toluidine Blue Staining and Transmission Electron Microscopy (TEM)

The regeneration axons were detected through facial nerve stem semithin section toluidine blue staining and facial nerve stem ultrathin section transmission electron microscopy analyses.

### 2.8. Data Collection and Statistical Analysis

Optical densities of GAP43 were measured using Quantity One software version 4.4.0 (Bio-Rad, Hercules, CA). For each rat, the number of surviving motoneurons was quantified by counting the number of neurons containing a visible nucleus-nucleolus in every second 20 *μ*m section throughout the length of the facial nucleus. Motoneuron counts were recorded as the percentage of motoneurons contralateral to the axotomy and graphed as the mean and standard error of the mean. One-way ANOVA was performed using Stata 8.0 software (Stata, College Station, TX) and *P* < 0.05 was considered significant.

## 3. Results

### 3.1. Following Reinjury, GAP43 mRNA and Protein in Facial Motoneurons Were Initially Upregulated, but Then Gradually Decreased

**Figure 1:**
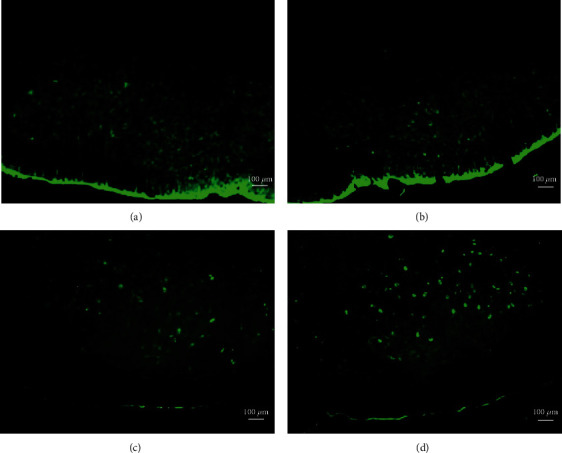
In Group I, GAP43 was expressed at a low level on the normal control side (a) and chronically axotomized side (b), but was higher in the facial nucleus at 12 w reaxotomy (d) compared with the control side (c).

Fluorescence labeling was used to identify cellular expression of GAP43 after facial nerve axotomy and reaxotomy. In facial nerve motoneurons of Group I animals (axotomy only), GAP43 was expressed at a low level on both the control side (Figure 1(a)) and the chronically axotomized side (Figure 1(b)). However, GAP43 expression gradually decreased on the reinjured side. Animals with reaxotomy at 12 w exhibited higher GAP43 expression in the ipsilateral facial nucleus (Figure 1(d)) compared with the control side (Figure 1(c)). At 28 w after the initial axotomy, GAP43 expression in the reinjured side was not higher than the control side and may have been weaker.

**Figure 2:**
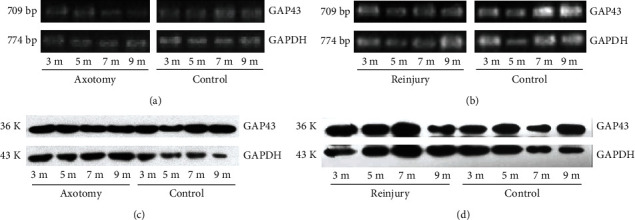
RT-PCR and western blot analysis of total RNA. GAP43 transcripts and GAP43 protein expression at 3, 5, 7, and 9 months after axotomy.

GAP43 mRNA transcripts were semiquantified by RT-PCR analysis of total RNA purified from facial nuclei from five independent experiments. In Group I animals (axotomy only), GAP43 transcripts in the axotomized side were present at a lower level compared with control sides at 12, 20, 28, and 36 w after axotomy (Figure 2(a)). However, as observed by microscopy, GAP43 mRNA expression was upregulated in injured sides compared with control sides of Group II animals reaxotomized 12 w after the initial facial nerve axotomy. However, GAP43 mRNA was present at a similar level to that of control sides when reinjury was performed at 20 w or 28 w. At 36 w, GAP43 transcript levels were lower in injured sides compared with control sides (Figure 2(b)).

Western blot analysis was used to semiquantify the expression of GAP43 protein, which was visualized as a 36 kDa band. At all experimental time points in Group I (axotomy alone), GAP43 protein was lower in the injured side compared with the control side (Figure 2(c)). In Group II (reaxotomy), GAP43 protein expression was increased when reaxotomy was performed 12 w or 20 w after the initial facial nerve axotomy. Expression of GAP43 protein was similar to that of its control side at 28 w, but was decreased at 36 w (Figure 2(d)).

### 3.2. Facial Nerve Axons Initially Regenerated and Then Gradually Decreased in the Reinjured Side

When reaxotomy was performed at 12 w or 20 w, toluidine blue staining and transmission electron microscopy revealed regeneration of facial nerve axons on the reinjured side. At 28 w, there was a significantly reduced number of regenerating axons (data not shown).

**Figure 3:**
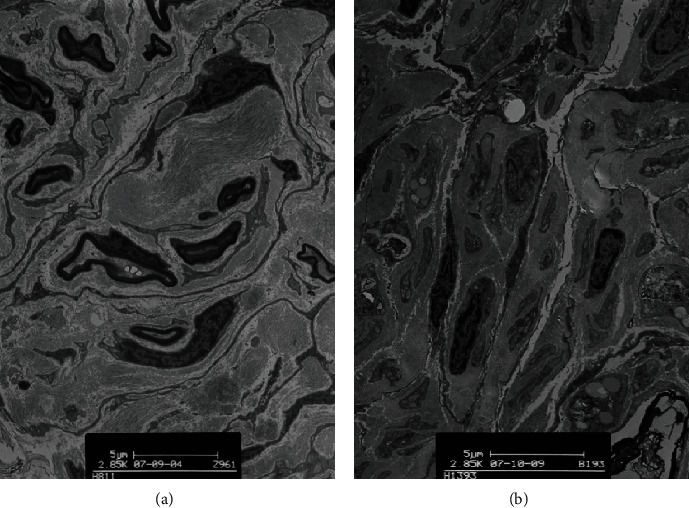
Most of the region was filled with collagen fibers and perineuria appeared normal at 12 w (a). Transmission electron microscopy showed hardly any surviving axons were observed and perineuria appeared collapsed at 28 w (b).

Using transmission electron microscopy, regenerating axons and a small number of Schwann cells could be visualized. Most of the region was filled with collagen fibers, and perineuria appeared normal. However, hardly any surviving axons were observed and perineuria appeared collapsed at 28 w (Figure 3).

### 3.3. The Relationship between Changes in Facial Nerve Regeneration Potential and Shh

#### 3.3.1. Shh Is Expressed in GAP43-Positive Neurons and Not Glial Cells

**Figure 4:**
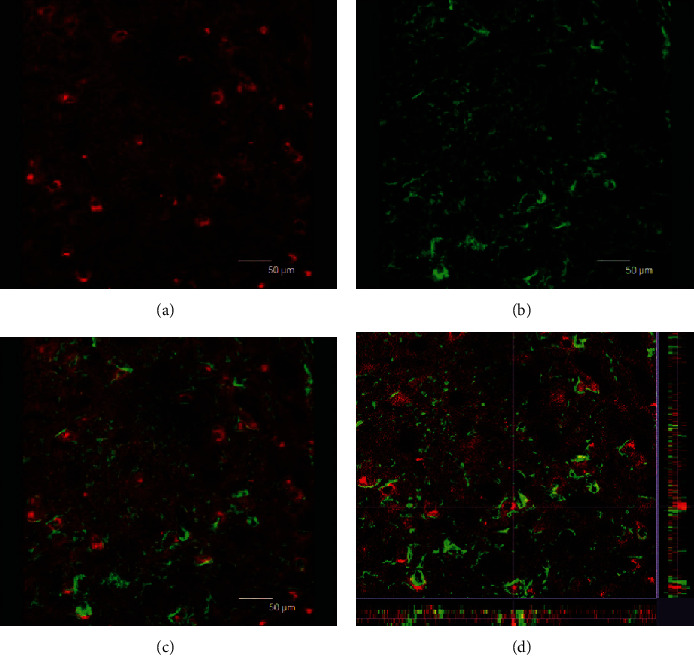
Facial nucleus double fluorescent-labeling laser scanning confocal fluorescence microscopy: red indicates sonic hedgehog-positive cells (a); green indicates GFAP-positive cells (b). GFAP-positive cells did not express sonic hedgehog. Panel (c) was the merger of panel (a) and panel (b). Panel (d) is the 3D image of confocal imaging (c).

**Figure 5:**
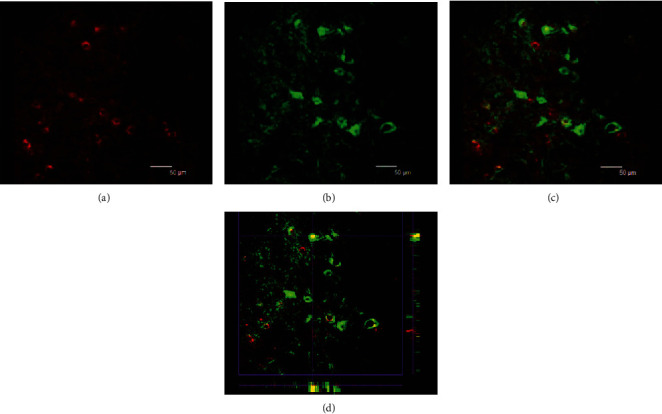
Facial nucleus double fluorescent-labeling laser scanning confocal fluorescence microscopy: green indicates GAP43-positive neurons (b); red indicates sonic hedgehog-positive cells (a). The GAP43-positive neurons were stronger expressing sonic hedgehog. Panel (c) was the merger of panel (a) and panel (b). Panel (d) is the 3D image of confocal imaging (c).

Double fluorescence labeling of the brain stem containing bilateral facial nuclei and subsequent laser scanning confocal fluorescence microscopy showed that although GFAP-positive glial cells did not express Shh, most GFAP-positive cells were located close to or wrapped around Shh-positive cells (Figure 4). GAP43 is a marker of neurons, and GAP43-positive cells in the facial nerve indicate motoneurons. These motoneurons expressed Shh at a higher level than GFAP-positive cells (Figure 5). Moreover, our results suggest that glial cells are activated after reinjury of the facial nerve, whereby they mainly locate around Shh-positive motoneuron cells.

#### 3.3.2. Following Facial Nerve Reinjury, Shh Protein Expression Decreased Over Time

**Figure 6:**
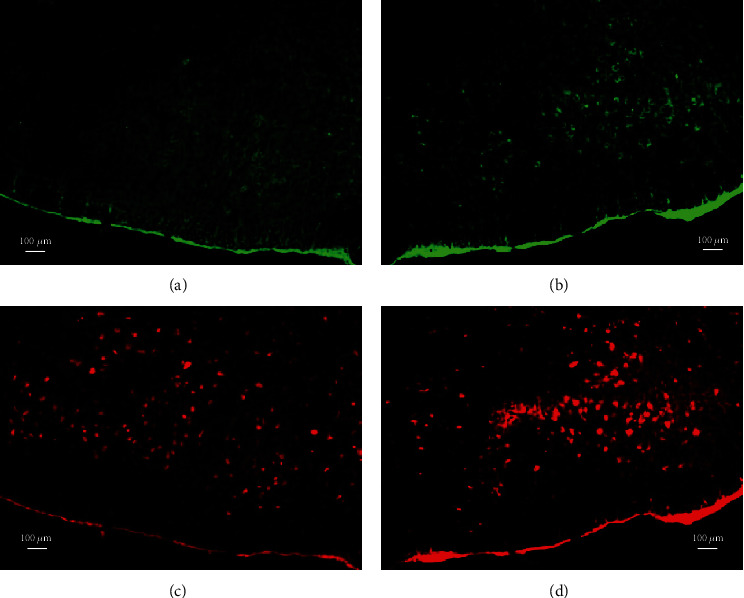
Double fluorescent labeling. In the reinjury group (16 w), expression of GAP43 (b) and sonic hedgehog (d) was apparently higher in the reinjured side compared with the control side of the facial nucleus (a) and (c).

In Group I single axotomy animals, double fluorescence labeling of the facial nucleus revealed weaker expression of GAP43 and Shh in the axotomized side compared with the control side (data not shown). In Group II reinjured animals, expression of GAP43 and Shh in the reinjured side was higher at 12 w compared with the control facial nucleus side (Figure 6). Reinjury at 36 w after the initial axotomy resulted in GAP43 and Shh expression levels that were no higher than observed in their respective control sides. After 36 w, the expression of GAP43 and Shh had decreased even more.

## 4. Discussion

Facial nerve axotomy in adult rats results in the degeneration of a third of facial motoneurons [33]. The loss of neurons takes several weeks and it is not understood how and why the remaining two-thirds of facial motoneurons survive. However, it is known that neurons have the potential to regenerate for a certain period following facial nerve axotomy [34, 35].

Factors that affect axonal fate after facial nerve axotomy include slow retrograde transport of large molecules such as cytokines and trophic factors, as well as the loss of target-derived trophic factors [34, 36, 37]. Genetic factors may also be associated with the potential to regenerate.

Very often, facial paralysis patients cannot be operated on immediately, which means that surgical procedures to repair the facial nerve can occur long after the initial paralysis. The time frame during which reparative surgery is still effective is unclear. Facial nerve false neuroma near the brain stump needs to be removed prior to facial paralysis transplant surgery. After this initial removal, nerve transplantation is carried out. Whether and the extent to which the facial nerve regenerates is dependent on its regeneration potential after facial nerve stump removal. If the capacity for facial nerve regeneration is extremely low, repair is difficult, even after the nerve graft has been performed.

The results of this study suggest that facial nerve regeneration mainly occurs after early reinjury of the chronically axotomized facial nerve. Therefore, the regeneration potential of the facial nerve is related to the timing of the second axotomy. Interestingly, only early reinjury (occurring less than 5 months after the initial injury) induced upregulation of Shh and Smo, whereas later reaxotomy had no effect.

Strong Shh immunoreactivity was observed in the cell bodies of facial motoneurons (GAP43-positive cells), but not detected in the cell bodies of astrocytes (GFAP-positive cells). This selective upregulation of Shh in reaxotomized motoneurons may play an important role in altering its functions. The regeneration of motoneurons may be dependent on Shh, which may influence regeneration through (as yet) unidentified molecules. Additional studies of Shh signaling are required to clarify precisely how the cellular network is preserved in nerve regeneration.

Our study also showed that Shh was upregulated in a time-dependent manner. Three months after facial nerve axotomy, Shh in the facial nucleus on the experimental side was significantly lower compared with the control side. Following facial nerve axotomy, motoneurons of the facial nucleus do not have a target; therefore, we hypothesize that the observed decrease in Shh results from the loss of a target. However, as nearly a third of facial motoneurons were lost and lower expression of Shh could simply be caused by the absence of neurons.

Our experiments show that the later the time point of reinjury, the weaker the Shh expression and at the same time the weaker the GAP43 expression. We speculate that maybe because of the decrease in Shh activation capacity may lead to a reduction in facial nerve regeneration. It is well known that astrocyte responses occur around neurons after facial nerve injury; astrocytes were closely related to neuronal cells and regulate the development and repair of the central nervous system. A variety of cytokines secreted by astrocytes also play an important role in regulating the signal transmission and synaptic transmission [5], as further confirmed in this study.

We propose that after facial nerve axotomy, the function of residual motoneurons is different. The surviving motoneurons are in a substate, and although parts of the motoneurons are still viable, they have very poor ability to regenerate nerve fibers. We speculate that as Shh signaling is activated, downstream regulatory transcription factors play an important role in the reversal of these substate neurons and can subsequently stimulate nerve regeneration.

We also found that at 4 months after facial nerve axotomy, facial nerve perineuria had disaggregated and a few myelin sheaths could be detected. However, 5–7 months after the initial axotomy, the perineurium of the nerves had disappeared and the remaining spaces had been replaced by fibrous connective tissue. This is another reason why axotomized facial nerve surgery should be carried out as early as possible.

Clinically, for some patients who cannot undergo surgery soon after facial nerve injury, surgical treatment in the later stage, including facial nerve decompression, could be regarded as facial nerve reinjury. Our results have a therapeutic implication for clinical treatment of facial nerve after injury and imply that regeneration can be promoted through transgenes. We have shown that after initial axotomy, there is a critical time period that determines the potential of facial nerve regeneration after initial axotomy, and that activation of the Shh signaling pathway is closely related to facial nerve regeneration after reinjury.

## 5. Conclusion

The purpose of this study was to investigate the nature of facial nerve regeneration to better understand the critical time points and mechanisms involved in regeneration. After facial nerve chronic axotomy in rats, the regeneration potential of the facial nerve peaked within 5 months and maybe was potentially dependent on activation of the Shh signaling pathway.

## Data Availability

The data used to support the findings of this study are included within the article.
